# Kojic Acid as a Natural Hypopigmenting Agent: Biological Activities, Delivery Challenges, and Nanotechnology-Based Strategies

**DOI:** 10.3390/pharmaceutics18080933

**Published:** 2026-07-29

**Authors:** Maryam A. Elbekbashy, Reham S. Elezaby, Maha Nasr, Gehanne A. S. Awad

**Affiliations:** 1 Department of Pharmaceutics and Industrial Pharmacy, Faculty of Pharmacy, Ain Shams University, Cairo 11566, Egypt; 2 Department of Pharmacology and Therapeutics, College of Medicine and Health Sciences, United Arab Emirates University, Al Ain 15551, United Arab Emirates

**Keywords:** drug delivery systems, inorganic nanocarrier, kojic acid, lipidic nanocarrier, polymeric nanocarrier

## Abstract

Hyperpigmentation is a multifaceted process that involves the overproduction and uneven distribution of melanin. The number of patients with skin hyperpigmentation is on the rise, representing the most common complaint in dermatology clinics. Treatment focuses mainly on melanocyte reduction and stopping melanosome production due to their expected degradative effects. Kojic acid (KA), a widely known natural depigmenting agent of fungal origin, acts by inhibiting the action of tyrosinase, the enzyme that catalyzes melanin production. Although it has been used for many years in skin-lightening preparations, kojic acid suffers from many problems that reduce its efficacy, including poor chemical stability, hydrophilicity, and adverse effects. Many modern systems like polymeric, lipidic, and inorganic nanocarriers have been used to provide controlled release, improve the delivery of kojic acid to the target site of action, and provide protection during storage. Consequently, the aim of this narrative review is to present the current literature reports concerning the biological activity of kojic acid, with particular emphasis on recent advances in nanotechnology-based delivery systems, including lipidic, vesicular, polymeric, inorganic, and other emerging nanocarriers developed for pharmaceutical and cosmetic applications. This review also introduces prospective, next-generation delivery strategies that remain unaddressed in the current literature, which may optimize the therapeutic efficacy of kojic acid. It also reports the main challenges that face the use of nanoparticles for kojic acid delivery, namely, the lack of clinical validation, limited safety data, and large-scale production reproducibility, as well as regulatory constraints, thereby identifying key research gaps and future priorities that necessitate the prioritization of clinical evaluation of kojic acid delivery systems.

## 1. Introduction

Topical cosmetic–pharmaceutical hybrids are a group of compounds intended to elicit health-positive effects with cosmetic benefits by affecting the skin’s biological function, hence presenting a link between personal-care products and pharmaceuticals. One of their commonly used applications is the treatment of hyperpigmentation [1].

Normal pigmentation in adults is a way of protecting human skin from the harmful action of UV radiation. However, melasma and solar lentigo, as well as skin cancer, are among the skin problems that may occur due to overproduction of pigment [2]. Hyperpigmentation, also medically known as hyper-melanosis, is defined as excessive melanin production primarily related to a higher number of melanocytes due to sunlight exposure, secondarily following treatment with certain drugs, or accompanying uncommon reasons causing melanocyte hyperactivity, like hormonal imbalance, inflammation, or any stimulus affecting the melanocyte stimulating hormone (MSH) [3]. Melanocyte activity and melanin production can also be affected by fibroblast growth factor, which is a hormone that acts on the renal proximal tubules to hinder phosphate resorption and calcitriol fabrication, while causing perturbation in skin pigmentation and in tyrosinase enzyme activity [4]: the key enzyme in melanin production.

Hyperpigmentation can generally be divided into four groups: post-inflammatory hyperpigmentation, melasma, solar lentigines and drug-induced pigmentation. The term “post-inflammatory hyperpigmentation” (PIH) has come into increasing use in recent years to describe skin darkening following skin healing from several inflammatory conditions such as burns, radiation therapy, laser treatment, wounds, psoriasis, atopic dermatitis, and acne. These conditions are characterized by abnormally high melanin synthesis. After being squeezed, acne lesions can darken and become more pigmented. PIH can be further subdivided into epidermal and dermal PIH, where epidermal PIH fades more rapidly than dermal PIH. PIH usually appears as irregular patches, ranging in color between yellow-brown in epidermal PIH and blue-black in dermal PIH, preceded by erythema and located in areas of previous inflammation or injury [5,6].

Melasma is a chronic acquired hyperpigmentary disorder in which brown macules with irregular borders are symmetrically distributed in photo-exposed areas, especially on the face, which causes psychosocial embarrassment that greatly impacts quality of life [7]. It affects all races but has higher prevalence in darker skin tones (Fitzpatrick skin phototypes III-V) and also affects women more than men due to hormonal factors (sex hormones, pregnancy). Melasma is subdivided into three predominant facial patterns: (1) centrofacial, the most prevalent pattern, mainly affecting the forehead, nose and upper lip, (2) malar, which only affects the malar cheeks of the face, and (3) mandibular, which mainly affects the jawline and chin [8,9].

Solar lentigines, commonly known as “sun spots” or “age spots”, are benign macular hyperpigmented lesions resulting from increased melanin production in the epidermis in response to chronic UV sunlight exposure. They mainly appear in photo-exposed regions like the hands, arms, face and neck, with incidence rates increasing with increasing age and most reported cases being in individuals above the age of 50 [10,11].

Hyperpigmentation may also occur following consumption of tetracyclines, antimalarials, nonsteroidal anti-inflammatory drugs, amiodarone, and antipsychotic drugs, a condition known as “drug-induced pigmentation”, affecting the skin, mucosal surfaces, nails, and hair [12,13].

Melanin is produced through a process called melanogenesis, initiated by tyrosinase-mediated oxidation of L-tyrosine to dopaquinone, the substrate for melanin synthesis [14]. The main site of tyrosinase production is melanocytes, followed by storage and transport via specialized organelles called melanosomes, pigment-containing structures within melanocytes. This melanosome insulation protects other cell organelles from oxidation during melanin formation [15].

The global cosmetic market and industry, especially the skin product domain, demonstrate a huge interest in natural products and their applications. The commonly used anti-hyperpigmentation molecules are presented in Table 1 [16,17].

The hypopigmenting effects of different molecules can be assessed using various approaches, namely, mushroom tyrosinase assays, cell culture assays, animal studies, and human clinical studies. Mushroom tyrosinase enzyme inhibition is an in vitro, cheap, and simple test used in early, high-throughput, fast screening of possible anti-hyperpigmentation molecules. It is also helpful in measuring the inhibition potency of each molecule as well as identifying the type of inhibition (competitive, non-competitive, etc.) through kinetic studies [38]. However, despite these advantages, it suffers from several drawbacks; for instance, several studies reported large differences between different molecules in inhibition patterns and values for mushroom tyrosinase and human tyrosinase, possibly producing misleading results. Moreover, since it is an in vitro test, it fails to evaluate the ability of drug molecules to penetrate the cellular membrane bilayer to perform their action [39].

Cell culture-based evaluation has also been widely used to assess the depigmenting activity of many molecules. In vitro cultured melanocytes have been widely used to measure melanin content and tyrosinase activity, which is very useful in testing whitening molecules. Among the most commonly used cell lines are mouse melanoma B16 cells, whose genomic composition is very similar to that of human epidermal melanocytes, with the same mechanism of melanogenesis [40]. Moreover, they are easy to culture, maintain pigmentation due to their high melanin production capacity, and respond to activation by α-MSH [41,42]. However, B16 cell lines cannot consistently predict human outcomes, as there are still biological differences between them and human skin, thereby necessitating careful interpretation of the results [43].

Animal models like mice (the most commonly used animal model) and zebrafish have been used to test the depigmenting effects of many molecules. Zebrafish are small-sized, easy to handle, and have higher reproduction rates than mice. They also show high efficiency of drug penetration through the skin, allowing direct observation of melanin changes on the surface of the skin without complicated experimental procedures, and they exhibit great similarities with human skin, making them great candidates for studying anti-hyperpigmentation molecules [44].

Although animal studies provide cost-effective, accessible subjects to conduct research under highly controlled conditions, the findings can be misleading because of differences between animals and humans in physiological responses and properties such as skin thickness, hair follicle density, and immune response [45,46]. These challenges make it harder to apply animal findings to humans, thereby necessitating human-based clinical studies. Only human-based clinical trials can determine whether tested molecules reduce hyperpigmentation and, if so, to what extent, as well as delineate their safety profiles [47]. However, conducting an impactful clinical trial faces many obstacles such as high costs, ethical and regulatory requirements, sometimes lengthy timelines, and difficulty in recruiting and retaining participants for the whole period of the trial without any dropouts [48].

Among the aforementioned molecules, kojic acid (KA) is one of the most widely used agents on the market for the management of pigmentation disorders. It is an organic acid produced as a secondary metabolite by a variety of fungi such as *A. oryzae*, *A. candidus*, *A. flavus*, and *A. effusus* through aerobic fermentation [49]. This compound was first discovered and isolated from the mycelia of the fungus *Aspergillus oryzae* grown on steamed rice in Japan by Saito. Later on, the name kojic acid was introduced by Yabuta from the Japanese term “koji”, which means steamed rice [50].

**Figure 1:**
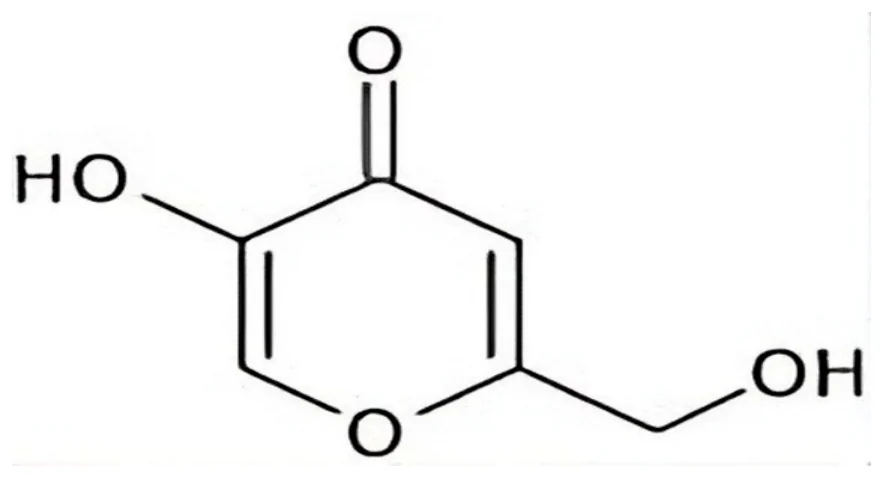
Chemical structure of kojic acid.

KA, also known as 5-hydroxy-2-hydroxymethyl-4-pyrone (Figure 1), is produced from different substrates with 2–7 carbon atoms, where the highest yield of KA is achieved when using glucose as the carbon source [51]. It was proposed that the biosynthetic pathway involves the direct conversion of glucose through a multistep process that involves the action of several enzymes like glucose-6-phosphate dehydrogenase, gluconate dehydrogenase, and hexokinase [52]. Over the years, research revealed that KA biosynthesis by *A. oryzae* requires a KA gene cluster made of three genes, namely, kojA, kojR, and kojT, where kojR is sandwiched between the other two genes [53]. A group of scientists tested the effects of kojR and overexpression of the entire gene cluster on KA synthesis [54]. They concluded that overexpression of kojR considerably increased the expression of kojA and kojT, thus significantly increasing the KA yield. Moreover, other researchers have proposed the involvement of genes such as AoKap1, AoKap2, and AoKap4 [55,56,57], ZRT [58], AoGld3 [59], and HirA [60]. For many years, KA has been used in the Japanese market as a functional food. In addition, due to its antioxidative and color-preservation properties, it has been a key player not only in the cosmetic industry but also in the food industry [61].

**Figure 2:**
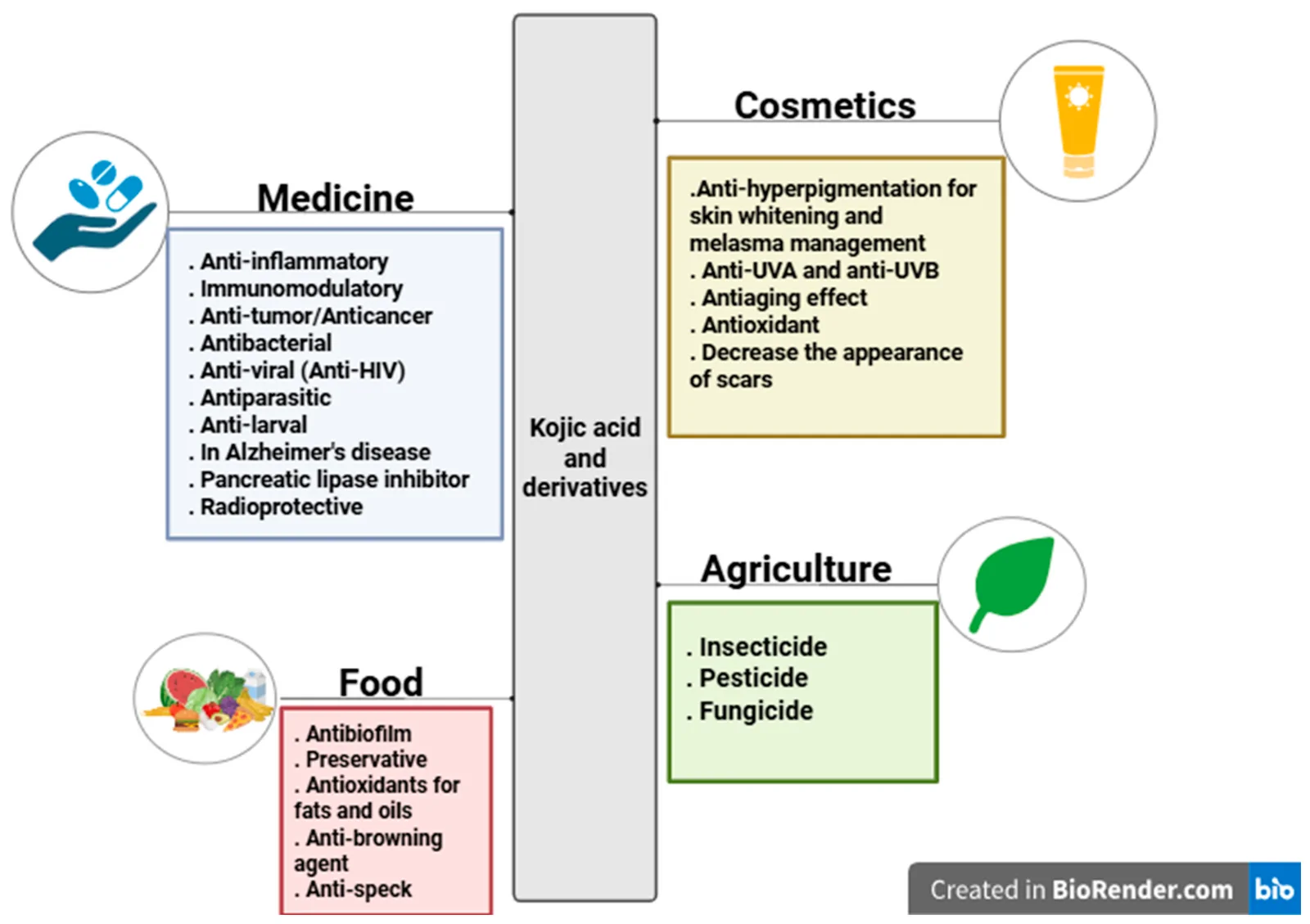
General applications of KA and/or derivatives. Created using BioRender. Elbekbashy, M. (2026) https://BioRender.com/jviimvy.

Despite the abundance of favorable applications of KA (Figure 2), its topical absorption is mainly limited by its solubility characteristics (Table 2), in addition to its poor storage stability and possible skin sensitization [62,63,64]. Despite these limitations, KA remains one of the most widely investigated depigmenting agents because of its well-established efficacy as a tyrosinase inhibitor, favorable biological profile, and relatively low cost compared with many newer skin-lightening agents. Moreover, KA possesses additional antioxidant, anti-inflammatory, antimicrobial, and emerging therapeutic activities, extending its potential beyond cosmetic applications. Importantly, most of its shortcomings, including poor stability, hydrophilicity, limited skin penetration, and irritation potential, are formulation-related rather than intrinsic pharmacological limitations. Therefore, KA derivatives, like kojic acid dipalmitate, kojic acid monooleate, and kojic acid monolaurate, or amino acid derivatives, have been proposed to overcome various challenges [65,66,67,68,69]. Nanotechnology also paves the way for better delivery and effectiveness of loaded cargoes [70]. Therefore, the aim of this review is to examine the biological activity and nanotechnology solutions for KA and its derivatives to overcome their delivery challenges. Unlike previously published review articles that primarily focused on the biological activities, cosmetic applications, or individual nanocarrier systems of KA, the present review provides an integrated overview linking biological activities with current and emerging nanotechnology-based delivery strategies.

## 2. Methodology

This narrative review adopted a comprehensive approach to collect and analyze studies investigating nanotechnology-based carriers used to deliver KA and its derivatives to overcome their delivery limitations and improve therapeutic outcomes. Studies were collected from three international databases, Scopus, PubMed, and Google Scholar, using targeted keywords such as “kojic acid”, “kojic acid derivatives”, “kojic acid dipalmitate”, “nanoparticles”, “advanced delivery systems”, “vesicular nanoparticles”, “lipid-based nanoparticles”, “polymeric nanoparticles”, and “metal-based nanoparticles”. The search was limited to studies published between the years 2000 and 2025, and only studies in English were included to ensure understanding and consistency. Lastly, to ensure high-quality evidence, the inclusion criteria focused only on the peer-reviewed literature, prioritizing experimental studies that revealed measurable improvements in drug delivery and providing a robust foundation for evaluating nanoparticles as delivery systems for KA.

## 3. Cosmetic Applications

### 3.1. Antimelanogenesis

**Figure 3:**
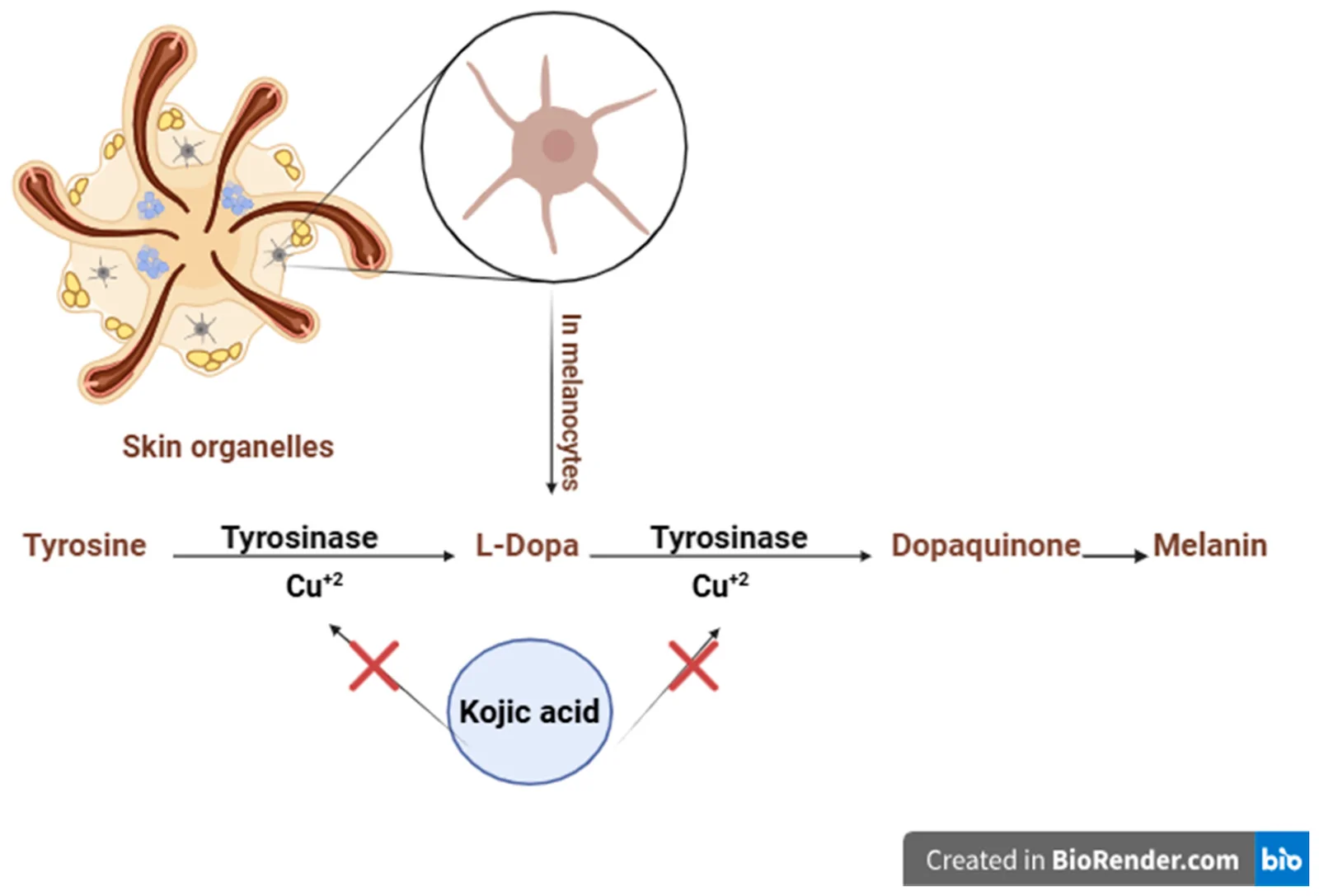
Tyrosinase inhibition mechanism of KA. Created using BioRender. Elbekbashy, M. (2026). https://BioRender.com/kq4pcjp.

Hyperpigmentation encompasses various disorders like post-inflammatory hyperpigmentation, melasma, and others [78,79]. Tyrosinase, a copper-containing enzyme, catalyzes the reaction that converts the amino acid tyrosine into L-Dopa (Figure 3), which is rapidly converted to dopaquinone, eventually turning into melanin [80]. As a tyrosinase inhibitor, KA is believed to reduce melanin synthesis by chelating copper ions at the enzyme binding site (Figure 3) [70,81,82]. Another study reported that the hypopigmenting effect of KA is associated with induced interleukin-6 production in keratinocytes following testing in human melanocyte/keratinocyte co-culture [83]. Moreover, KA was shown to inhibit the activation of NF-ƙB in a human keratinocyte cell line, suggesting a new pathway that might be involved in its hypopigmenting activity [84]. Also, an in vivo zebrafish model showed that the expression of melanogenesis mediators, microphthalmia-associated transcription factor (MITF), tyrosinase, and tyrosinase-related proteins 1 and 2 (TRP-1 and TRP-2) was downregulated upon testing a newly synthesized KA derivative [85]. The concentration of KA in cosmeceutical marketed products ranges between 0.2 and 1%. Some reports do not recommend exceeding its concentration more than 1% to avoid the possibility of irritation, inflammation, rash, itchiness, and pain, while others report its safety up to 2% [86,87]. Some animal studies report the carcinogenicity of KA upon systemic absorption, with no similar reports related to topical use [88].

Several studies assessed the activity of KA and compared it with other well-established anti-hyperpigmentation molecules. For instance, a clinical trial established the effectiveness of 1% KA in treating hyperpigmentary conditions like melasma, PIH, and age spots [89]. Another clinical study comparing the efficacy of 4% hydroquinone (HQ) cream and 0.75% KA cream following a once-daily application for a period of 12 weeks revealed the faster response and higher overall efficacy of 4% HQ as a hypopigmenting agent. However, it is worth mentioning that HQ can cause exogenous ochronosis, a black discoloration of skin that is hard to reverse. Consequently, its use was banned in many countries, and the FDA imposed several restrictions, suggesting the higher safety of KA [90,91,92]. Another randomized clinical study showed that the use of a combination of 1% KA and 2% HQ showed better results in the management of melasma compared to the use of each molecule on its own [93]. Also, a meta-analysis of published clinical trials showed that KA, tranexamic acid, cysteamine, and azelaic acid showed comparable efficacy in the topical treatment of melasma, with cysteamine and azelaic acid being the most irritant [94]. In another report, it was stated that thiamidol and tranexamic acid were superior to KA in safety, making them better candidates for melasma management [95]. A double-blinded clinical trial was also conducted to compare a 2% KA cream and a combination of 10% glycolic acid and 2% HQ, finding that 2% KA was effective in the management of melasma without causing serious complications [96]. A 12-week randomized study tested KA for skin lightening in the management of dyschromia, showing that vitamin C/kojic acid showed better efficacy in improving skin tone and radiance compared to HQ [97].

### 3.2. Reactive Oxygen Species (ROS)-Related Biological Activity

Prolonged skin exposure to sunlight causes coarse and fine wrinkles, decreases skin elasticity, causes dryness, and elicits hyper- or hypopigmentation spots in a process known as skin photoaging [98]. Excessive ROS production due to ultraviolet radiation (UVR) exposure results in antioxidant imbalance, causing DNA damage, inflammation, and upregulation of matrix metalloproteinases (MMPs), leading to collagen fiber degradation [99]. In addition, oxidative stress promotes melanogenesis by stimulating the expression and activity of melanogenic mediators, including tyrosinase, thereby contributing to UV-induced and post-inflammatory hyperpigmentation. Therefore, antioxidants such as KA may complement their direct tyrosinase inhibitory activity by scavenging ROS, reducing oxidative stress, and indirectly suppressing melanin overproduction. Furthermore, UVR-induced oxidative stress releases iron from its binding proteins, catalyzing more ROS release [100]. As both a metal chelator and a free radical scavenger, a 5% KA solution was reported to prevent skin wrinkling, thereby contributing to considerable photoprotection in a UV-irradiated animal model by controlling ROS activity [101]. A hypothetical study using quantum chemistry calculations revealed that the enol moiety of KA is more important for its antioxidant activity than the alcohol group [102]. Regardless, it is important to note that no human clinical data are currently available on the photoprotective activity of KA.

Furthermore, increased ROS levels cause oxidative stress, leading to delayed and defective wound healing [103]. A research group suggested that using a 9% KA ointment for eight consecutive days could accelerate wound healing in wounded rats while improving their final appearance by limiting post-inflammatory hyperpigmentation [76]. However, the data were based on an in vivo study only, and no clinical study was performed.

## 4. Repurposing of Kojic Acid

Owing to the diverse biological activities of KA and its derivatives, they have been repurposed for use in different diseases, as highlighted in this section. However, it should be noted that the majority of these findings are based on in vitro experiments or preclinical animal models; therefore, they require further clinical validation before translation into therapeutic applications.

### 4.1. Antimicrobial Activity

The antimicrobial activities of KA and its derivatives have been well-documented [104,105]. KA extracted from *Aspergillus allahabadii* showed good antibacterial activity against both Gram-positive and Gram-negative bacteria [106], with better probable activity against the latter [107]. The disruption of the cell membrane’s integrity, causing potassium ions leakage leading to the dissipation of zeta potential, has been found to have a detrimental effect on bacteria [108]. In addition, KA showed antibiofilm activity against both *Aeromonas sobria* and *Staphylococcus saprophyticus* in a dose-dependent manner, suggesting that KA destroys extracellular polymeric substances secreted by microorganisms, reducing their pathogenicity [109]. The antibacterial activity of KA against *Escherichia coli* was also reported, where the catechol groups interact with cellular phospholipids, disrupting the cell membrane and promoting membrane permeability. Moreover, it was suggested that KA uses its chelating properties to form complexes with inter- and intracellular metal ions, thereby impairing ionic balance and eventually causing cell shrinkage and death [110].

### 4.2. Antiparasitic Activity

The cutaneous antileishmanial activity of KA was believed to result from macrophage activation, promoting superoxide anion production for parasite killing [111]. Furthermore, its anti-tyrosinase activity during in vitro culture of adult *Schistosoma mansoni* worms significantly decreased the laying of phenotypically normal eggs, thus providing a potential approach to eradicating schistosomiasis [112]. KA fungistatic activity against *Cryptococcus neoformans* was reported as well, where the inhibition of melanin produced by the fungus increased the fungus’s susceptibility to microbicidal peptides [113]. Similarly, a special effect was found in chromoblastomycosis, a chronic human subcutaneous fungal infection with excessive melanin production, where KA antimelanin activity guarded against fungal hostility to the immune system, thereby helping inhibit fungal growth in vitro [114].

### 4.3. Neurological Applications

Parkinson’s disease (PD) has been associated with neuromelanin, a dark brown intracellular pigment strongly related to neuropathology. The accumulation of neuromelanin has been found to initiate PD, highlighting the detrimental effects of the brain pigment despite its neuroprotective action. However, the potential participation of neuromelanin in PD remains obscure due to the lack of the pigment in laboratory animals [115]. The role of tyrosinase in the synthesis of neuromelanin was reviewed in 2022 by Nagatsu et al., who proposed a biosynthetic pathway similar to melanin in human skin and hair [116].

In 2024, Ali et al. showed that KA, by virtue of its anti-inflammatory and antioxidant effects, may suppress the neuro-inflammation and oxidative stress caused by lipopolysaccharides (LPSs) through the regulation of the TLR4/NF-κB signaling pathway in both the cortex and hippocampus of male wild-type mice brains, via three pathways. KA downregulated the expression of toll-like receptor 4 (TLR4), phospho-nuclear factor kappa B (p-NFκB), and phospho-c-Jun N-terminal kinase (p-JNK) proteins. Moreover, it increased the expression levels of the endogenous antioxidants nuclear factor erythroid-2-related factor 2 (Nrf2) and the enzyme hemeoxygenase 1 (HO-1), which were downregulated in the neuro-inflamed brain. Finally, KA inhibited LPS-mediated TNF-α and IL-1β production. These results were confirmed by improved learning behavior and spontaneous alternation in the Morris water maze and Y-maze tests, suggesting the ability of KA to heal short-term memory loss in the LPS model [117].

**Figure 4:**
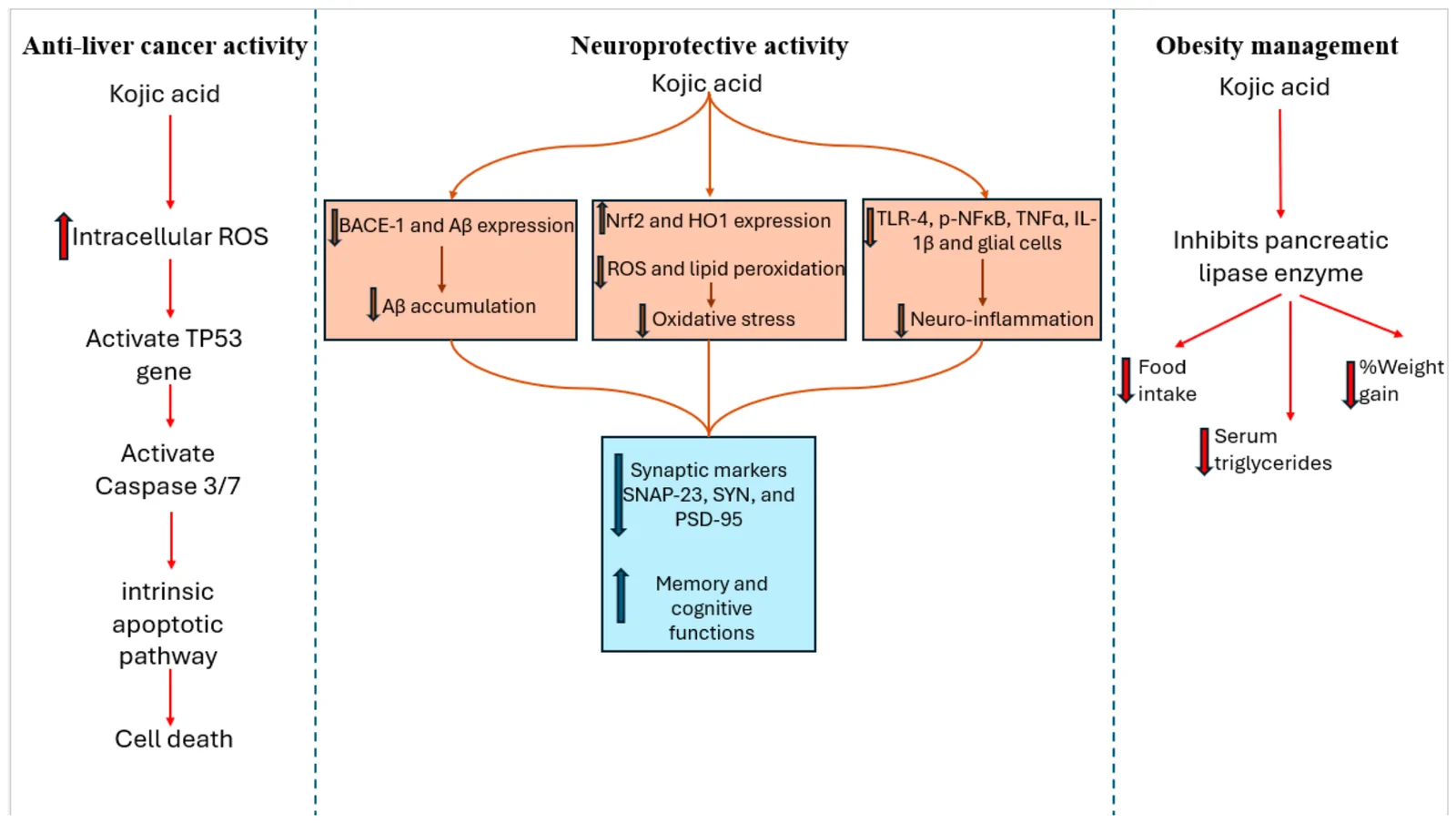
A schematic representation of KA’s most prominent biological activities. The pathways represent the principal molecular targets reported in the literature and are intended as simplified schematic summaries rather than exhaustive signaling cascades.

Likewise, an Alzheimer’s disease (AD) mouse model was used to test the antioxidative and anti-inflammatory effects of KA, where an oral treatment caused a reduction in brain oxidative stress by upregulating the expression of the Nrf2 and HO1 antioxidant genes [118]. It also reduced the expression of amyloid-beta (Aβ) and beta-site amyloid precursor protein cleaving enzyme 1 (BACE-1), thus reversing AD pathology. The synaptic markers SNAP-23, SYN, and PSD-95 were also enhanced by the use of KA. Moreover, downregulation of inflammatory cytokine expression, as well as a reduction in glial cell activity, would help in reducing neuro-inflammation (Figure 4).

Recently, in 2025, acetylcholinesterase (AChE) inhibition with an anti-aggregating effect for amyloid-β was determined for a series of heteroaryl thiol-linked KA derivatives. With proof of brain penetration, this natural class would possibly positively contribute to the management of AD. Previous researchers prepared KA-loaded mesoporous silica nanoparticles (MSNs) with anti-AChE activity, suggesting improved cognitive functions in AD patients [119,120]. An in vitro study using a KA dimer caused inhibition in amyloid-β (Aβ) deposition, along with metal chelation, reduced oxidative stress, and provided neuroprotection, suggesting a multi-armed AD treatment [121].

### 4.4. Obesity Management

After Orlistat, the only FDA-approved pancreatic lipase activity inhibitor, Elkorany et al. positioned KA as a possible second member of this group for obesity management. By combining an obesity-induced animal model, an in vitro lipase inhibitory assay, and in silico molecular docking tests, they suggested the KA interaction with key amino acids (Lys80, Trp252, and Asn84) in the pancreatic lipase binding site as a mechanism for activity inhibition, with an accompanying reduction in serum triglyceride levels (Figure 4) [122].

### 4.5. Ocular Applications

KA also exhibited anti-senescence activity in human corneal endothelial cells (HCECs), a cause of blindness by aging, through upregulation of p-NF-κB, downregulation of galectin 8, laminins, and P21 siRNA, and migration enhancement in senescent HCECs. KA also reduced HUVEC spheroid sprouting induced by senescent HCECs, thus alleviating senescence and angiogenesis [123].

### 4.6. Immunomodulatory Activity

The radioprotective and macrophage activation of KA were also delineated [124,125]. The pretreatment of C57BL/6 male mice with a single subcutaneous KA injection returned blood count parameters to their normal values, thus protecting them against ionizing radiation. This radioprotective effect has been correlated with the antioxidant nature of KA, which scavenged free radicals produced after radio-exposure [126]. Finally, KA was believed to act as an immunomodulatory agent by promoting the secretion of interleukin-6 cytokine, increasing the expression of EMR1-F4/80, a surface protein that acts as a mediator during immune responses, and reducing the expression of CD11b and CD14 mono-nuclear cell surface markers, which are downregulated during the differentiation of monocytes into macrophages, thus stimulating the differentiation of monocytes into macrophages [127].

### 4.7. Anticancer Activity

Recently, a study on HEPG2 hepatocellular carcinoma proved that KA derivatives triggered the intrinsic p53 apoptotic pathway, increasing the concentration of intracellular ROS and upregulating the pro-apoptotic TP53 gene; this gene prevents mutated cells from dividing, with caspase 3/7 activation (Figure 4) [128]. KA allomaltol derivatives tested against two different breast cancer cell lines (MCF-7 and MDA-MB-231) suggested increased passive delivery across the cell membrane mediated by enhanced lipophilicity. It stimulated overexpression of pro-apoptotic genes (TP53 and Bax) accompanied by suppression of anti-apoptotic genes (Mdm-2 and Bcl-2) in MCF-7 cell lines. Meanwhile, MDA-MB-231 cell death was caused by necrosis [129]. Recently, KA derivatives synthesized using “click linker” technology [130] induced apoptosis in breast and pancreatic cell lines by downregulating the anti-apoptotic protein ID1, inducing PARP cleavage, and eventually causing cell apoptosis. Worthy of note is that comparative studies evaluating the therapeutic selectivity of KA and its derivatives are largely lacking. Therefore, future investigations should include normal cell controls and in vivo toxicity assessments to establish the therapeutic window of these compounds.

## 5. Challenges of KA Delivery

Despite the wide spectrum of KA’s restorative qualities, it faces various obstacles that reduce its efficacy. The hydrophilic nature of KA (logP = −2.45), due to its two hydroxyl groups, limits its dermal absorption, ultimately minimizing its therapeutic efficacy [64,131,132]. Another major problem is its photo/thermosensitive behavior, causing its degradation in aqueous solutions [63]. Furthermore, the oxidation and chelation of KA result in the browning of its formulations and reduce its antimelanogenic activity [133]. Lastly, it has been reported that KA is highly unstable at pH values above 7 [75].

In addition, contact dermatitis, erythema, and a higher risk of sunburn in patients with sensitive skin might be encountered with topical application of KA. Moreover, KA degradation products containing a 4-pyrone moiety present a risk to human health and can be carcinogenic [134,135,136,137]. As previously mentioned, the topical use of KA at concentrations up to 1% is well tolerated by patients with no significant side effects [138,139]. KA’s shortcomings can be addressed by synthesizing derivatives with superior properties and/or making use of nanoparticulate drug delivery systems.

## 6. Overcoming KA Limitations

### 6.1. Fabrication of KA Derivatives

KA properties in cosmetics have been enhanced by reacting its alcoholic hydroxyl group with various chemical entities to convert it into esters, amino acid derivatives, hydroxyphenyl ethers, metal chelates, or tripeptide derivatives. Esters like kojic acid dipalmitate (KAD) or amino acid derivatives have been proposed to overcome various challenges [65,66,67,68,69].

KAD is a derivative hydrolyzed by esterase enzymes to release the active form in the skin. Its enhanced oil solubility boosts its affinity for the stratum corneum [140]. With enhanced oxidation, light, and heat stability, KAD is preferred in skin whitening formulations [141]. However, its increased lipophilicity would preclude direct drug incorporation in various formulations with the required dose, calling for modulated delivery systems [142]. Other KA esters, like kojic acid monooleate, monolaurate, and monopalmitate, were synthesized and tested for their efficacy and safety. The KA esters provided significantly lower cytotoxicity and similar anti-tyrosinase activity compared to KA, with a confirmed depigmenting ability [143]. A large number of Mannich bases of KA have also been synthesized, with a few members showing significant tyrosinase inhibitory activity [81]. Furthermore, the dimeric structure of KA was reported by Rho et al., and the thioether linkage produced a recognized anti-tyrosinase effect with nitric oxide (NO) production [144].

Kojicones A and B are two novel tricycle skeleton derivatives obtained from a fungal strain, *Aspergillus versicolor*. They inhibit NO production in murine RAW 264.7 cells with a decrease in mRNA expression of IL-6, IL-1*β*, TNF-*α*, and iNOS while stimulating IL-4 expression. Moreover, colitis was significantly controlled with a lowered disease activity index, anticipating potential anti-inflammatory effects [145]. A comparison between the most relevant KA derivatives is presented in Table 3.

### 6.2. Nanotechnology-Based Delivery Systems of KA and/or Its Derivatives

Nanomaterials are designed to provide site-specific targeting for enhanced bioavailability, alongside reduced adverse effects. Improved drug shelf life and in vivo stability are achievable, in addition to physicochemical property modulation, such as solubility [149,150]. The advantages and limitations of the most commonly used nanocarriers for dermal delivery are illustrated in Table 4. The nanomodalities for KA and derivative delivery are presented in Table 5. Non-tackled approaches are also included to enlighten the reader with the possible prospects of this molecule being repurposed in various fields.

#### 6.2.1. Carriers for Dermatological Applications

##### Vesicular Systems

**Figure 5:**
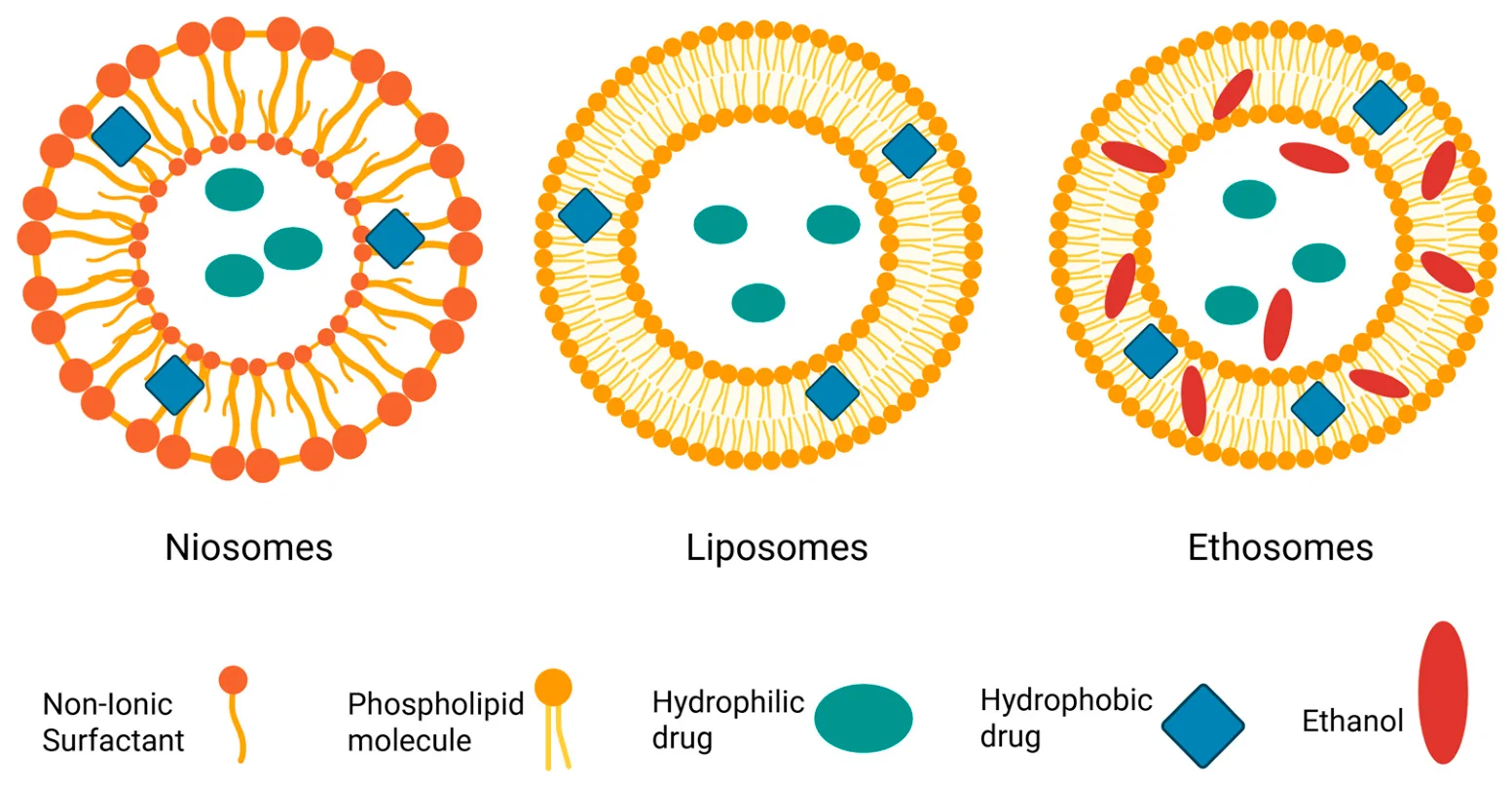
Some vesicular systems used for KA delivery. Created using BioRender. Elbekbashy, M. (2026). https://BioRender.com/ipfens3.

The self-assembly of amphiphilic molecules in water was the first step in the formation of vesicular systems with their known highly ordered structures. These systems can localize drug action at a specific site, avoiding unwanted side effects in other parts of the body. Vesicles can also be used for drug controlled release with zero-order kinetics to ensure steady drug levels [172]. They include liposomes, ethosomes, and niosomes. A diagram clarifying differences between some vesicular systems is shown in Figure 5.

##### Ethosomes

Ethosomes can achieve excellent permeation improvement and drug release. They are characterized as ultradeformable, helping in facile stratum corneum penetration with deeper skin deposition. Their high alcohol concentration reduces the lipid transition temperature (Tm) in both the stratum corneum layer and vesicles, thus fluidizing skin layers and increasing vesicle flexibility [173,174].

An ethosomal gel provided deeper and more sustained KAD delivery compared to a free KAD gel, with greater inhibition of melanin synthesis [175]. The improved skin elasticity accompanying higher water content supported ethosomes’ suitability for improving aged skin conditions.

##### Spanlastics

Spanlastics are surfactant-based nanovesicles prepared from a mixture of surfactants and edge activators that exhibit high deformability, allowing improved penetration of molecules into deeper skin layers [176]. In a preliminary report, KA spanlastics were formulated to control dark skin discoloration caused by psoriatic flare-up and restore uniformly toned skin [177].

##### Polymeric Nanoparticles

Carriers used in nanoparticle fabrication encompass natural, synthetic, and semisynthetic polymers. Natural polymers are subdivided into proteins, such as silk, collagen, gelatin, albumin, and polysaccharides, such as dextran, hyaluronic acid, alginate, and chitosan (CS) [178]. Synthetic polymers and their numerous subclasses are also extensively reported [179].

Examples of self-assembled vesicular amphiphilic molecules formed from block copolymers are polymeric nanoparticles (10–100 nm), biodegradable polymeric micelles, and polymersomes. Their hydrophilic chains surround a hydrophobic core, preventing its contact with the external aqueous phase. They also provide controlled drug release while protecting the encapsulated moiety against harsh external conditions [180,181]. Their self-assembly depends on the “hydrophilic fraction (f)” of the polymer. A polymer with “f” equal to 35 ± 10% is more likely to self-assemble into polymersomes, but if “f” is bigger than 50%, the aggregates will form micelles [182]. The main difference between micelles and polymersomes is that the former can encapsulate lipophilic drugs in their cores, while the latter can encapsulate both hydrophilic and lipophilic drugs in their cores and bilayers, respectively [183].

##### Polymeric Micelles

Saraei et al. prepared KA amphiphilic terpolymer-based polymeric micelles [184]. Firstly, KA was protected by benzoyl chloride at the OH group (BOP), which was esterified with the carboxyl group of methyl acrylate (MA) to give BOPMA. BOP also reacted with bromoisobutyryl bromide (BIBB) to give BOPBr. BOPMMA was then obtained by free radical polymerization between BOPBr and BOPMA, followed by esterification with N-phthaloylated CS to give the terpolymer poly [5-(benzyloxy)-4-oxo-4H-pyran-2-yl] methyl acrylate/polyacrylic acid/chitosan (PBOPMA-PAA-CS). The terpolymer containing KA polymeric micelles exhibited stronger antibacterial activity compared to both gentamycin and ampicillin. The same group synthesized KA-containing acrylates and evaluated their antifungal and antibacterial effects [185].

##### Polymer Conjugates

In one study, CS reacted with chloro-kojic acid in dimethylsulfoxide or dimethylformamide to form chit/koj1,6 covalent conjugates for film formation [186]. In another study, solution polymerization was used to incorporate KA into poly(carbonate esters), polyesters, and natural diacids to enhance KA stability, which is released by polymer hydrolytic degradation. The chemical nature of the polymer backbone had a great effect on the degradation process [133]. In addition, in a US patent, KA was contained in a poly(carbonate ester) degradable polymer to enhance KA stability and control its release. Various polymer precursors accommodating different kojic acid–linker–kojic acid monomers were used to adjust polymer properties. Polymers containing groups in their backbone provided KA upon the backbone’s degradation [187].

#### 6.2.2. Carriers for Cosmetic Applications

##### Niosomes

Similar to liposomes, niosomes are self-assembled nanovesicles prepared with nonionic surfactants instead of PL and thus share liposomes’ advantages. The lack of PL provides an added benefit of oxidation avoidance, justifying their improved stability and storage conditions with reduced production costs [188,189]. Firstly developed by L’Oréal in the 1970s for cosmetic use [190], niosomes are regarded as promising systems for the delivery of KA in the management of hyperpigmentation. Due to their lipophilicity and surfactant permeation-enhancing activity, niosomes will promote intimate skin contact, improving KA penetrability. KA niosomes (kojisomes) were prepared by Saeedi et al. using an “environmentally friendly method” without the use of organic solvents. A histopathological examination revealed that the application of a Kojisomal gel did not cause cutaneous irritation, in contrast to a drug solution, and helped in deeper skin layer penetration [191].

#### 6.2.3. Dual Functioning Carriers

##### Lipidic Nanocarriers

In addition to their similarity to the lipid membrane, the lipophilicity of lipid-based nanocarriers facilitates their molecular interaction with the stratum corneum, thereby promoting effective dermal penetration. Recently, they have been considered effective chemical enhancers, opening a gate in the skin barrier for small hydrophilic molecules like KA to target the melanocytes found in the basal cells of the skin epidermis (stratum basale). They also enhance the stability of the loaded molecule.

##### Nano- and Microemulsions

Nanoemulsions (NEs) and microemulsions (MEs) are liquid dispersions prepared by amphiphile-driven stabilization of a water and oil mixture [192]. Despite having droplet sizes in the nano-range, NEs and MEs differ in their physical properties and thermodynamic stabilities. An ME is a spontaneously formed, thermodynamically stable system that maintains a high level of stability, usually obtained by high surfactant concentrations (>10%), thereby leading to a generally smaller particle size than an NE. On the other hand, an NE is kinetically stable and thermodynamically unstable, so it usually requires high energy input for preparation and tends to break down over time [193,194]. MEs are prepared using low-energy methods such as the spontaneous emulsification method, while NEs can be prepared using both low- and high-energy methods [195,196].

Enhanced safety of KA monooleate (KMO) O/W NEs compared to the oil itself was reported [197]. In another report, enhanced antibacterial activity against *S. aureus* was also reported for KA ester (KAE) NEs compared to the free ester, with the in vivo toxicity profile tested on embryonic zebrafish displaying normal development without any observed deformation [198].

KAD was combined with rosehip oil, rich in fatty acids and with restorative skin structure properties, in an NE to improve the stability and activity of both ingredients [142].

##### Solid and Solid/Liquid Matrix Lipid-Based Carriers

**Figure 6:**
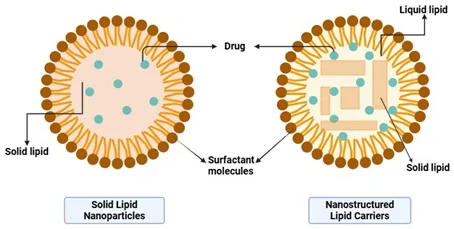
Solid lipid nanoparticles versus nanostructured lipid carriers. Created using BioRender. Elbekbashy, M. (2026). https://BioRender.com/sf8bze3.

SLNs are formed of solid lipids stabilized by a surfactant layer [199,200,201]. Their main advantage is their easy scale-up [202]. Despite their nanosize and numerous advantages, SLNs suffer from particle growth and drug expulsion during storage due to perfect crystallization [203,204]. As a solution, a new generation of lipid nanoparticles was developed, known as second-generation nanostructured lipid carriers (NLCs) [205,206]. The addition of a liquid lipid to the solid lipid core favored higher drug loading while augmenting stability during storage and accelerating drug release. The difference between SLNs and NLCs is illustrated in Figure 6.

KA solid lipid microparticles prepared by the hot homogenization method using carnauba wax as the sole lipid with 1% Tween 80 were not capable of containing sufficient KA (5–15%) in their matrix [207]. Meanwhile, KAD was successfully loaded in SLNs [208].

Compared to free KA, KA SLNs enhanced percutaneous delivery and tyrosinase inhibitory activity [132]. However, higher KA entrapment in NLCs was obtained with the addition of oleic acid, providing a lipidic core with less crystalline abilities. The loading of KA in NLCs potentiated both the anti-tyrosinase and antioxidant activities of KA [64].

##### Liposomes

Liposomes are vesicular systems made of an aqueous core surrounded by a phospholipid (PL) bilayer to which cholesterol is usually added to enhance membrane rigidity and decrease its permeability to solutes [209,210,211]. The use of liposomes in cosmetics provides many benefits, among which is their skin-hydrating effect. The occlusive layer they form on the skin improves the penetration of encapsulated molecules into the skin and guards skin cells from sun and sweat [212]. Moreover, in damaged dry skin, empty liposomes will react with dermal components to enhance the protective effect of the stratum corneum [213]. Al- Edresi et al. reported an improvement in insoluble KAD loading capacity in liposomes using an active loading technique [214].

Dipalmitoylphosphatidylcholine or DPPC KA liposomes coated with N-(2-hydroxyl) propyl-3-trimethyl ammonium chitosan chloride (HTCC) show reversed particulate charges with a higher zeta potential value and improved skin penetration [215]. Moreover, gelatinized core liposomes were fabricated to overcome stability problems and low hydrophilic drug entrapment, as well as boost the antibacterial activity of KA against methicillin-resistant *Staphylococcus aureus* (MRSA) infection [216]. The higher core viscosity reduced KA leakage, helping in drug encapsulation. Furthermore, MRSA infection was adequately suppressed by targeting and activating the monocytes at infection sites.

##### Polymeric Nanocarriers

Following an ex vivo skin deposition test and melanin content assessment, collagen–CS nanoparticles revealed improved penetration of KA into deeper skin layers with ameliorated antimelanogenic activity, suggesting the suitability of this system for cosmetic use. The deeper skin deposition can be ascribed to the nanoparticle size, hair follicle targeting, and the interaction between the positive charge provided by CS and the negatively charged stratum corneum, forming microstructure cavitations in the stratum corneum and thus promoting dermal absorption [217].

In a non-cosmetic study, a group of scientists tested the use of KA-loaded silk fibroin (protein synthesized by silkworms) nanoparticles against *Leishmania amazonensis* [218]. These nanoparticles enhanced KA antileishmanial activity, with a reported reduction in IC_50_ from 27 to 7 μg/mL against amastigotes. Moreover, the system induced apoptosis-like cell death of the parasite by causing organelle dysfunction and perturbation of cellular processes.

##### Polymeric Nanocapsules

KAD rosehip nanoemulsions were encapsulated in various polymers like Eudragit RS100, Eudragit S100, poly(ε-caprolactone), and PLGA75/25 to yield highly stable nanocapsules, which were recommended for potential use in cosmetic formulations [69].

##### Inorganic Nanocarriers

Metal and metal oxide inorganic nanocarriers were prepared based on silver, iron, gold, cerium, and copper metals. Metal oxide-based systems like zinc oxide or iron (III) oxide were also synthesized to improve the reactivity and efficiency of their corresponding metal-based systems [219].

Mesoporous silica nanoparticles (MSNs) have been recently explored for skin drug delivery and cosmetics. Formed by silicon dioxide, they can assemble a network structure generated from the spatial arrangements of a large number of pores. Their appealing large surface area shows different shapes and sizes and huge pore volumes with modifiable pore sizes, making them attractive for drug delivery and cosmetics. Surfactants of different sizes were used as soft templates for MSN production, where larger pore sizes were induced by long-chain surfactants, while shorter-chain ones yielded smaller-sized pores [220,221,222]. Likewise, MSNs have the ability to control drug delivery in response to predefined physical, chemical, or biochemical stimuli by using pore-blocking molecules known as “gatekeepers”, thus preventing the premature release of encapsulated drugs [223,224].

Functionalized MSNs containing 3% KA were prepared by Lima et al. [119]. Despite the low drug concentration, the nanosystem successfully showed high antibacterial activity against *C. albicans*, *S. aureus*, *S. enterica*, and *B. cereus*, with promising AChE and tyrosinase inhibitory activity. The system was also capable of protecting loaded KA against enzymatic degradation.

Seeking to improve KA MSN loading, Andrade et al. used 3-aminopropyltriethoxysilane-functionalized silica nanoparticles (MSNAPTES-KA5) and reached a 12% drug loading [120]. The increase in KA content markedly potentiated AChE and tyrosinase inhibitory activity in addition to antimicrobial activity against *C. albicans* and *S. aureus.* The higher AChE inhibition emphasizes the potential of KA in AD, but further in vivo and clinical investigations are still required to prove its therapeutic efficacy.

Iron oxide magnetic nanoparticles (MNPs) were also used as KA carriers and were coated with either CS or PEG to form smart KA nanocomposites [225]. In another report, both CS- and PEG-coated KA nanocomposites exhibited anticancer activity against melanoma cells compared with free KA [226].

MSNs have been used in several published studies to deliver many molecules like antioxidants and antiaging drugs for cosmetic use, where they protect loaded drugs against degradation and increase their dermal permeation. In addition to being biocompatible, they can also be surface-modified by molecules like cell-penetrating peptides, which can lead to deeper skin penetration. However, other problems like their long-term safety and industrial scalability need to be addressed in depth [171]. On the other hand, MNPs have been widely studied for their potential in the biomedical field rather than cosmetic use owing to their possible toxicity, where they can induce inflammatory responses, oxidative stress, and cell membrane perturbations; therefore, despite their potential in cosmetics, they should be used with care [227].

##### Miscellaneous Systems

This section encompasses heterogeneous formulation platforms that cannot be readily classified under the previously discussed lipidic, vesicular, polymeric, or inorganic nanocarriers. Although structurally diverse, these systems have been investigated as alternative strategies to improve the topical delivery and therapeutic performance of KA and its derivatives.

Due to their distinctive lamellar structure, which provides outstanding viscoelasticity and extended moisturizing properties, liquid crystals (LCSs) have attracted the attention of the cosmetic industry for their potential use in a variety of conditions. They improve preparation stability, especially liquid formulations, enhance skin hydration, and impart a smooth skin sensation to the products containing them [228]. Gonçalez and co-authors prepared KA-loaded liquid crystals (LCSs) to regulate drug permeation through the skin, thus improving its activity [229]. Decreasing the percentage of water in LCSs increased permeation through the stratum corneum, with higher retention in the epidermal and dermal layers in an in vitro skin permeation and retention test. Such behavior may be attributed to reduced drug solubility, freeing KA to move through different skin layers. The in vitro cytotoxicity test also proved KA-loaded safety, hence suggesting it as a promising platform for the topical delivery of KA.

Although the loading of KAD in a nano-cream formed of emulsion droplets in the nano-range did not significantly impact the drug’s release, it was able to increase KA penetration into the skin as well as prolong the residence time in the skin from 3 to 7 days through accumulation in hair follicles [66]. Furthermore, a W/O/W multiple emulsion that was also loaded with KAD had less product cell toxicity and enhanced in vitro antioxidant activity for a longer period of time, pinpointing its possible efficiency for skin aging [230]. Finally, a chemical stability study was performed to test the stability of a KA-loaded emulgel prepared by Khadivi et al., where the stability of the emulgel was tested at different temperatures (5, 25, 30 and 40 °C), with/without sunlight exposure over a period of three months. The emulgel was able to reduce the degradation of KA compared to a simple gel, thus enhancing the stability of KA. Moreover, a significant reduction in drug content was observed in emulgels stored at higher temperatures with sunlight exposure, highlighting the importance of storing emulgels in opaque packaging at refrigeration temperature (5 °C) [231]. Among the miscellaneous delivery systems discussed, emulgels and LCSs seem to be the most practical platforms for topical delivery of KA owing to their ease of formulation, improved skin retention, controlled drug release, and favorable patient acceptability.

## 7. Future Perspectives: Unexplored Delivery Platforms for Kojic Acid and Its Derivatives

Given the comprehensive examination of KA and its derivatives and their numerous applications in medical treatments and cosmetics, we would also like to suggest delivery methods that have not yet been deployed. Despite their variability, the literature on KA and its derivatives still has not met the ultimate goal of overcoming KA delivery limitations, which is why we provide insights into possible futuristic delivery systems that can mitigate these delivery challenges.

KA-enhanced skin penetration via the use of CS-coated liposomes was highly appreciated in another publication [138]. Although liposomes are the most commonly used vesicular system in the cosmeceutical field, similar phospholipid-based approaches have been developed to allow enhanced skin penetration. Among these are phytosomes, invasomes, hyalurosomes, transfersomes, glycerosomes, and others [232]. Deeper skin layer penetration can be achieved with transfersomes, which can squeeze themselves through the intercellular channels of stratum corneum cells for both medical and cosmetic purposes [233,234,235,236]. A lower dose of KA is possible with a longer residence time upon application, which can be achieved by hyalurosomes. They are modified vesicular systems in which sodium hyaluronate is coupled with phospholipids, revealing penetration-enhancing abilities and gelling properties [237]. In addition, hyaluronic acid provides hydrating, antioxidant, anti-inflammatory, and antiaging activities as well [238,239,240,241]. Furthermore, the incorporation of glycerol into phospholipid-based vesicles increases bilayer fluidity and enhances membrane permeation, giving rise to “glycerosomes” [242]. Glycerol, with humectant and emollient properties, is believed to augment the accumulation of different molecules in different skin layers [243,244].

Coupling phospholipids with penetration enhancers like terpenes and ethanol produces invasomes with enhanced vesicular flexibility. However, due to their potential for skin irritation, the choice of terpenes should be well regulated to avoid any possible harmful effects [245]. Terpenes are classified as generally regarded as safe (GRAS) by the FDA, presenting concentration-dependent safety; indeed, it has been reported that concentrations of terpenes below 5% have low irritation potential. In addition, decreasing ethanol concentrations were anticipated to inhibit any hypersensitivity reactions [246,247]. Phenylethyl resorcinol-loaded transfersomes and invasomes were prepared and compared to conventional liposomes. Both systems revealed stronger tyrosinase inhibitory activity, which may be attributed to greater accumulation in the deeper skin layer [248]. In another study on acne, invasomes showed a 2.5-fold increase in skin deposition compared to a drug solution, highlighting the superiority of the system [249].

Upon exposure to ultraviolet radiation, the level of antioxidants, including glutathione, increases to neutralize released ROS. A mixture of poly(lactide) (PLA) and redox-responsive poly (ethylene glycol)–*block*–poly(lactide) (PEG–*block*–PLA) containing a disulfide bond was synthesized, which released cargo by cleavage of the disulfide bond by glutathione, thereby creating a smart redox-responsive system that can selectively release drugs in a reducing medium [250]. Also, ROS-responsive MSNs successfully released glabridin in deeper skin layers, reversing photodamage and reducing hyperpigmentation [251]. In addition, CS-coated PLGA nanoparticles released ceramides in the skin in a pH-dependent manner, in which CS, degraded by the slightly acidic pH of the skin, successfully regenerated the stratum corneum in atopic dermatitis [252].

Fullerenes, made of carbon atoms with a hollow cage-like structure, show strong antioxidant properties that can protect the skin against UV-induced damage, thus further potentiating the anti-hyperpigmentation activity of KA [253]. Fullerenes are not absorbed by the skin, and animal-based experiments showed no irritation or skin sensitization following their topical application [254]. However, the safety of fullerenes in topical delivery should be studied in depth, as they have been reported to be phototoxic, as exposure to visible and ultraviolet rays produces singlet oxygen, which causes lipid peroxidation and cytotoxicity [255].

## 8. Challenges Facing the Use of Nano-Drug Delivery Systems in Cosmetics

The use of nanoparticles in cosmetics currently faces three main challenges: safety concerns, regulatory inconsistencies, and scale-up and manufacturing reproducibility. These challenges limit the clinical application of nanocarriers in cosmetics.

### 8.1. Safety Concerns

The high permeability of nanoparticles through biological barriers is a major advantage for drug delivery; however, it could be disadvantageous and lead to different toxicities, specifically when used in daily products [256]. It has also been proposed that the smaller the particle size, the larger the surface area-to-volume ratio will be, which subsequently increases the chemical reactivity and the risk of toxicity, meaning that the nanosize range can also be considered as a double-edged sword [257]. Lipid- and polymer-based nanocarriers are usually prepared from biodegradable and biocompatible starting materials, which makes them less hazardous than other nanoparticles, particularly metal-based nanoparticles [258]. For example, zinc oxide (ZnO) and titanium dioxide (TiO_2_) nanoparticles are commonly used in sunscreens. While most peer-reviewed papers stated that ZnO does not cross the stratum corneum, a bioassay utilizing human epidermal keratinocytes (HEKs) reported that 8–20 μg/mL of ZnO nanoparticles were internalized by the cells, leading to cytotoxic and genotoxic responses [259]. Several in vitro and in vivo studies have also shown that the use of TiO_2_ nanoparticles can induce genotoxicity through DNA and chromosomal damage, as well as in vitro gene mutations [260]. Currently, there is an obvious gap in research concerning the toxicological effects of nanoparticles used in dermo-cosmetics; accordingly, long-term in vitro and in vivo toxicological studies and bioaccumulation studies using different types of skin are of great importance to ascertain the safety of nanotechnology-based drug delivery systems in cosmetics.

### 8.2. Regulatory Aspects

To date, there is no clear worldwide accepted definition of nanomaterials used as cosmetic ingredients, and every country operates under its unique definition and regulatory system [154]. For example, the European Union (EU) ensures the safety of nano-cosmeceuticals through Cosmetics Regulation (EC) No. 1223/2009, in which article 2 specifies that all nano-ingredients should be registered through a free online portal known as the Cosmetic Products Notification Portal (CPNP) 6 months before introducing the product to the market, with proper assessment of safety and correct labeling by adding the word “nano” after the name of each nanomaterial in the list of ingredients [261]. The Scientific Committee on Consumer Safety (SCCS) assesses the safety of nano-ingredients based on detailed physicochemical characterization, exposure conditions and frequency, and toxicity profile of nano-ingredients as well as safety data [262]. In contrast to the EU, the USA does not require pre-marketing regulatory approval of nano-ingredients used in cosmetics (except dyes). Moreover, the FDA does not mandate manufacturers to disclose the presence of nano-ingredients, as it does not consider particle size to correlate directly with toxicity. Such an approach compromises transparency since consumers will not be aware of their presence in a product [263]. Also, the FDA does not require manufacturers to test their products for safety or use safety preclinical or human clinical studies, as it does not specify the type of tests required to test the safety of nano-cosmetics; mainly, the safety assessment is the manufacturer’s responsibility, where the FDA recommends characterizing ingredients by their physicochemical properties [264,265]. Therefore, regulatory and scientific communities need to agree on a single clear definition of nano-cosmeceuticals by establishing a standardized regulatory guideline that harmonizes nano-ingredient testing methods and sets clear standards for particle size polydispersity, morphology, and surface composition of decorated nanoparticles.

### 8.3. Manufacturing Reproducibility

One of the major challenges that nanotechnology-based systems face is their scale-up to the manufacturing level and batch-to-batch variation. The manufacture of nanosystems requires careful control over physicochemical properties of the system, like particle size, morphology, surface charge, encapsulation efficiency, and release rates, where small alterations could fundamentally alter the product’s safety, biological activity, stability, and therapeutic impact. Unfortunately, the scale-up usually alters the balance of production conditions like temperature, pH, shear rate, humidity, and others that control nanosystem properties, leading to batch-to-batch variation. Moreover, keeping nanocarriers stable with a homogeneous size distribution and preventing drug degradation over long periods is challenging, given their tendency to aggregate, crystallize, and oxidize under environmental stress [266,267]. Consequently, overcoming these challenges is crucial to upscaling nanosystems into good manufacturing practice (GMP)-compliant products ready for commercialization.

**Table 5:** Characterization of different drug delivery systems for KA and its derivatives.

System	Drug	Optimized Formulation Parameters	Route of Administration	Major Outcomes	Reference
Nanoemulsion	KMO	Castor oil with low comedogenicity and high ricinoleic acid content was used where the optimum formula was monodispersed with a PDI of 0.25, a PS of 110.01 nm, and a relatively high zeta potential value of −43.60 ± 0.23 mV, confirming particle repulsion.	Topical	- Nanosized particles at values below 200 nm were maintained at 25 °C and 4 °C but not at higher temperatures. - Safer KMO NE with less cytotoxicity compared to the plain oil.	[197]
	KMO	Using a central composite design, it was found that longer shear time caused a PS increase due to enhanced particle collision. Meanwhile, higher speed in a high-shear homogenizer decreased the PS. When using a magnetic stirrer, a maximum stirring speed of 271.77 rpm was needed. An 8.04 min shear time at 4905.42 rpm in a high-shear homogenizer prepared NE with a PS of 103.97 nm.	Topical	- Less toxic optimized formula had an IC50 > 500 μg/mL, indicating safety of NE. Also, eyrosinase inhibition of NE was higher than free KMO.	[268]
	KAE	NE with a PS of 110 nm was prepared using high- and low-energy emulsification techniques.	Topical	- KAE NE permeability increased from 4.94% at 1 h to 59.64% at 8 h. - Improved antimicrobial activity. -Reduced toxicity (LC50 > 500 μg/mL) when tested on zebrafish embryos, confirming the safety of KAE NE.	[198]
	KAD	It was possible to raise drug loading up from 1 and 2 mg/mL, but at 3 mg/mL, precipitation occurred. Both formulations had close PS, ZP, and pH values.	Topical	- NE needed storage under refrigeration. - KAD delivered to dermis, allowing melanocyte targeting without systemic side effects. - The scale-up process proved leakage of the drug at 2 mg/mL NE, while the 1 mg/mL succeeded in keeping the drug at the same concentration as in a laboratory experiment.	[142]
Microemulsion and microemulgel	KA	Oleic acid or caprylic/capric (CP) triglyceride-based MEs were developed. CP ethanol ME solubilization efficiency was the highest and showed the fastest release.	Topical	- KA ME prepared with CP triglycerides and ethanol as a cosurfactant effectively enhanced flux. - Four mechanisms were proposed for enhanced delivery, including high drug loading abilities, supersaturation caused by microemulsion phase transition upon skin application, and intimate contact of the ME with the skin due to extremely low surface tension, clarifying the superiority of the fluid formula over the emulgel and the penetration enhancement activity of the ME. - Conversion of the ME to a gel reduced the transdermal flux but showed superior flux to the aqueous solution.	[73]
Solid lipid microparticles	KA	The highest formula yield (87.7%) was produced by 10% KA homogenized at 15,000 rmp for 5 min.	N.D.	- Single lipid (carnauba wax) reduced encapsulation due to crystallization (5–15%). - Higher stirring speed increased entrapment. - Slow and progressive KA release.	[207]
Solid lipid nanoparticles	KAD	Among the glyceryl esters used, glycerol monostearate had the smallest PS (70 nm).	Topical	- Overall, 47% KAD loading. - Slower zero-order release. - Better penetration of the stratum corneum, leading to greater accumulation in the epidermis.	[208]
	KA	The combination of glyceryl monostearate, cholesterol, Span 60, and Tween 20 gave the smallest PS (156.97 nm) and the highest ZP (−27.67).	Topical	- Overall, 59% KA entrapment. - Controlled release. - Potentiated tyrosinase inhibition. - Enhanced percutaneous accumulation, achieving successful skin targeting.	[132]
Nanostructured lipid carriers	KA	The addition of oleic acid to glyceryl monostearate and cholesterol increased drug loading.	Topical	- Oleic acid raised the entrapment to 76.4%. - Enhanced skin permeation led to greater KA accumulation in the skin. - Reduced IC50 from 18.3 to 5.4 μg/mL. Improved tyrosinase inhibition.	[64]
Liposomes	KAD	Active loading in DSPC/cholesterol-based liposomes.	N.D.	- A concentration gradient was used as a driving force to improve KAD loading capacity in liposomes from 0.61 to 28.12%.	[214]
Chitosan (CS)-derivative-coated liposomes	KA	Coating DPPC/cholesterol liposomes with a CS derivative (HTCC) reversed their charge from −6.8 to 29.2 mV.	Topical	- Coating improved the fusion efficiency between positively charged liposomes and negative stratum corneum. - Higher penetration of KA into the skin, confirming the superiority of cationic liposomes as penetration enhancers. - Enhanced ability of melanin synthesis prevention.	[215]
Gelatinized core liposomes	KA	Gelatin added to the liposomal core reduced the PS and increased KA entrapment from 35.68% to 50%.	I.V.	- Loaded liposomes overcame antibiotic resistance by constant zero-order release. - Gelatin adsorbed on the liposomal surface was thought to act as an opsonic agent with specific binding abilities to monocytes via mannose receptors, thus enhancing monocyte targeting. -Vesicular particle size enhanced KA activity by enhancing phagocytosis.	[216]
Ethosomal gel	KAD	Smaller phosphatidylcholine concentration (3%) and higher ethanol concentration (35%) increased the loading of KA in ethosomes up to 90%. Ethanol at 35–40% produced the smallest ethosome size (134 and 148 nm). A rise in ethanol concentrations increased particle zeta potential, hence enhancing their stability.	Topical	- Ethanol, a permeation enhancer, improved the ethosomal gel flux and increased KAD deposition in deeper skin layers compared to the control gel. - Enhanced melanin, erythema, and sebum reduction. - Increased skin moisture level and elasticity.	[175]
Niosomes	KA	Increasing the cholesterol concentration to 1% increased both the PS (325 nm) and KA entrapment (39.341%).	Topical	- Increasing the cholesterol-to-SAA ratio decreased niosome permeability, thus raising KA encapsulation. - The niosomal gel exhibited a first-order extended release with deeper skin deposition and stronger suppression of melanin synthesis.	[191]
Spanlastic vesicles	KA	Vesicles formed from Span 60, along with nonionic surfactants. The obtained vesicles showed a PS of 234.2 ± 1.65 nm, with high entrapment efficiency (87.4% ± 0.84%) and significant sustained drug release.	Topical	- In vivo studies proved anti-psoriatic activity, with minimal changes in the expression of mRNA of cytokines. - An alternative for anti-psoriatic systemic therapy was proposed. - In silico study of KA docking in anti-psoriatic drug targets was also tried.	[177]
Collagen(CN)–chitosan (CS) nanoparticles CSCN	KA	Increasing the collagen amount to 500 mg increased the PS to 404.23 nm and KA entrapment to 82.34%.	Topical	- Positively charged CSCN-NP improved NP transfer across the stratum corneum to the inner dermal layer, leading to higher KA dermal accumulation (29.16%) in comparison to a KA HPMC gel. - Reduced melanin content to a lower level compared to the simple gel.	[217]
Silk fibroin nanoparticles	KA	KA silk fibroin nanoparticles with a PS of (176 nm) and a ZP of (−32.3 mV) were successfully obtained for Leishmania amazonensis.	N.D.	- The loading of KA into nanoparticles reduced the KA IC50 against L. amazonensis amastigotes from 27 to 7 μg/mL. - Increased selectivity towards amastigotes, improving the leishmanicidal activity of KA.	[218]
Polymeric micelles	KA	KA-derived terpolymers (kojic acid/acrylic acid/CS) were successfully used to fabricate micelles with a small PS (20 nm).	N.D.	- More effective antimicrobial activity (PBOPMA-PAA-CS) against *S. aureus* than against *E. coli*. - Stronger inhibitory effect compared to gentamycin and ampicillin, making them great candidates for future use in nanomedicine.	[184]
Nanocapsules	KAD	The charges of the nanocapsules varied according to the polymer used; it was positive with Eudragit RS100 and negative with other anionic polymers.	Topical	- Among the polymers used, PLGA kept the nanoemulsion stable for 180 days regarding KAD content. - The nanocapsules reach deeper skin layers in in vitro permeation.	[69]
Functionalized mesoporous silica nanoparticles (MSNs)	KA	KA was immobilized on MSN and MSN modified by aminopropyltriethoxysilane (APTES). Particles showed a PDI of 0.3, a PS of 218 nm, and a ZP of + 31.3 mV.	N.D.	- KA loaded at 3% still preserved its AChE inhibitory (66.28%), antibacterial, and tyrosinase inhibitory (28.57%) activities.	[119]
	KA	Soaking of functionalized nanoparticles in a KA solution with a higher concentration (5000 μg/mL) increased loading to 12%, with a PDI of (0.3) and a PS of (230 nm).	N.D.	- Higher KA loading increased the AChE inhibitory activity from 66.28% to 85.93%, suggesting its potential use in Alzheimer’s. - Increased tyrosinase inhibitory activity from 28.57% to 60.2%, revealing enhanced melanin synthesis suppression. - Improved *C. albicans* and *S. aureus* inhibitory activity.	[120]
Nanocomposites	KA	CS-coated iron oxide nanoparticles showed higher KA loading (12.2%) than PEG-coated nanoparticles (8.3%).	N.D.	- Nanocomposites slowed down the release of KA. - The tested formulae showed minimal to no antimicrobial activity because of the low drug loading.	[225]
	KA	The zeta potential of MNPs was −11.4 mV, and that of KA-Cs-MNPs and KA–PEG–MNPs was +14.2 and −25.8 mV, respectively.	N.D.	- KA in the nanocomposite system enhanced its anticancer activity against melanoma cells with no cytotoxicity to normal cells.	[226]
Liquid crystalline systems (LCSs)	KA	A formula made of 20% water, 30% cetostearyl isononanoate (oil), and 50% ethoxylated and propoxylated cetyl alcohol (surfactant) was chosen due to superior bioadhesion and controlled release of KA.	Topical	- The developed formulations could be manipulated, where changing the water or surfactant content could modulate the permeation of KA into the skin. - In vitro cytotoxicity assay of KA-loaded and KA-unloaded LCSs did not show any cytotoxicity.	[229]
Nano-cream	KAD	The nano-cream had a PS of <350 nm, while that of the normal cream was >5 μm.	Topical	- The release of KAD from nano-creams showed no significant variation from normal creams. Nonetheless, nano-creams effectively delivered KAD to the skin’s hair follicles, keeping it in the skin for a longer time, thus prolonging its activity.	[66]
Nanogel	KA	The optimized nanogel exhibited a smaller PS (746.48 nm) and a higher ZP (−67.1 mV).	Topical	- The optimized formula showed the highest percentage of drug release in a controlled manner with greater stability.	[269]
Multiple emulsion (W/O/W)	KAD	The multiple emulsion showed a large average PS of 1.534 μm and a low ZP of −13 mV.	N.D.	- The addition of KAD changed the flow behavior of the multiple emulsion from shear-thinning to Newtonian. Moreover, KAD’s toxicity was reduced while preserving its antioxidant activity.	[230]
Emulgel	KA	The optimal emulgel formulation containing 1% KA, 0.25% sodium metabisulphite and stored at 5 °C showed the highest KA retention after 3 months storage.	Topical	- The emulgel improved KA retention after 3 months of storage compared to the simple gel, proving its superiority for topical use. - To further protect KA against degradation, it is recommended to use the optimal antioxidant concentration and store at refrigerator temperature in a light-protected package.	[231]

AChE: acetylcholinesterase; DPPC: dipalmitoyl phosphatidylcholine; DSPC: 1,2-distearoyl-sn-glycero-3-phosphocholine; HPMC: hydroxypropyl methylcellulose; KA: kojic acid; KAD: kojic acid dipalmitate; KAE: kojic acid ester; KMO: kojic acid monooleate; ME: microemulsion; MNPs: magnetic nanoparticles; MSNs: mesoporous silica nanoparticles; N.D.: not disclosed; NE: nanoemulsion; PEG: polyethylene glycol; PLGA: poly(lactic-co-glycolic acid); PS: particle size; SAA: surfactant; W/O/W: water in oil in water; ZP: zeta potential.

## 9. Conclusions

Hyperpigmentation disorder evokes a lot of interest nowadays due to its psychological impact on patients. Despite the long list of whitening agents, KA remains one of the molecules with confirmed efficiency, though it suffers from stability and sensitivity problems. Its derivatives with enhanced stability and activity have addressed these drawbacks, but their safety has not been thoroughly assessed. The various applications of this natural product in depigmentation and as a free radical scavenger delineate it and its derivatives as star ingredients in skin-lightening and anti-aging products. Herein, we present different systems reported to improve dermal absorption and protect against degradation, thus potentiating its biological activity. Possible systems that can be used in the future for the topical delivery of KA or its derivatives were also suggested to enhance pharmacological outcomes and reduce side effects. In the case of KA, nanodelivery strategies should primarily focus on overcoming its intrinsic limitations, including poor chemical stability, hydrophilicity, and limited skin penetration, to maximize its effectiveness in topical cosmetic and dermatological applications. For KA derivatives, which are particularly lipophilic derivatives, such as KAD, that already exhibit improved physicochemical characteristics, nano-controlled-release delivery systems would be more beneficial. Furthermore, since some KA derivatives have demonstrated pharmacological activities beyond depigmentation, including antimicrobial, anti-inflammatory, neuroprotective, and anticancer effects, their potential for broader therapeutic applications following further mechanistic and clinical validation can be an interesting future perspective. However, to date, these systems are mostly investigated at the preclinical stage, and to the authors’ knowledge, none of these systems have reached the clinical study phase. This could be attributed to the absence of standardized protocols for comparison of different nanosystems, as well as hurdles related to large-scale manufacturing, long-term stability, and regulatory approvals. Moreover, no data were collected regarding the long-term safety profile of these systems. This research gap highlights the need for standardized skin permeation models, comparative efficacy studies, detailed irritation and long-term safety evaluations, and well-designed clinical trials to establish the therapeutic potential of these systems. Future efforts should focus on formulation optimization, scalable manufacturing, regulatory assessment, and clinical validation to facilitate the successful translation of KA and derivative-based nanocarriers from laboratory research to pharmaceutical and cosmetic applications.

## Figures and Tables

**Table 1:** A list of commonly used molecules present on the market as cosmetic agents for the management of hyperpigmentation.

Hypopigmenting Agent	Origin	Mechanism of Action	References
Alpha-arbutin (α-arbutin)	Isomer form of natural arbutin (glycosylated hydroquinone) biosynthesized by microorganisms or microbial glycosyltransferase. Arbutin is naturally produced in berry-producing plants.	- Inhibits the activity of tyrosinase enzyme, preventing the oxidation of L-dopa and melanin production. - Shows stronger anti-tyrosinase activity than normal arbutin and β-arbutin.	[14,15,18]
Ascorbic acid (vitamin C)	Water-soluble vitamin commonly found in citrus fruit.	- Blocks copper at the tyrosinase active site, hence reducing melanin production.	[19,20]
Azelaic acid	Dicarboxylic acid naturally produced in grains like rye, barley, and wheat.	- Inhibits the activity of tyrosinase enzyme. - Arrests the proliferation and differentiation of melanocytes by suppressing DNA synthesis.	[21,22]
Bakuchiol	Phenolic monoterpene, a major constituent of *Cullen corylifolium* seeds.	- Inhibits the α-melanotropic hormone that plays an important role in increased melanin production.	[23]
Cysteamine	Aminothiol synthesized in human cells as a byproduct of coenzyme A degradation.	- Inhibits the activity of tyrosinase enzyme. - Suppresses peroxidases while increasing glutathione content intracellularly and regulates copper and iron needed in melanin pathway synthesis.	[24,25,26]
Glabridin	Prenylated isoflavan extracted from *Glycyrrhiza glabra* L. (licorice).	- Downregulates the transcriptional/protein expression of tyrosinase, microphthalmia-associated transcription factor (MITF), tyrosinase, tyrosinase-related protein 1 and 2 (TRP-1 and TRP-2), and melanocyte-stimulating hormone receptor (MC1R).	[27,28]
Glycolic acid	Small two-carbon α-hydroxy acid.	- Exhibits combined anti-inflammatory, keratolytic, and antioxidant effects. - Engenders desquamation by disrupting cell adhesion, thus leading to fast pigment dispersion. - Inhibits the activity of tyrosinase enzyme.	[29,30]
Hydroquinone	Naturally occurring in coffee, tea, wheat, germ, pears, and others.	- Reversibly inhibits tyrosinase enzyme and selectively degrades melanosomes and melanocytes.	[31,32]
Kojic acid	Organic acid produced by fungi of Aspergillus species.	- Inhibits the activity of tyrosinase enzyme, reducing melanin synthesis.	[33]
Niacinamide	Nicotinic acid amide and one of the two principal forms of water-soluble vitamin B3.	- Slows down melanosome transfer from melanocytes to keratinocytes.	[34]
Retinoids	Vitamin A derivatives.	- Inhibit tyrosinase activity. - Inhibit melanosome transfer. - Accelerate shedding of melanin-filled keratinocytes.	[35]
Tranexamic acid	Synthetic analog of lysine amino acid, which is primarily used as an antifibrinolytic agent.	- Reduces plasmin levels by decreasing the activity of plasminogen activator, eventually reducing melanin production in melanocytes. - Competitive inhibitor of tyrosinase enzyme.	[36]
Thiamidol	Isobutylamido-Thiazolyl-Resorcinol, discovered upon the screening of 50,000 Thiazolyl-Resorcinol derivatives.	- The most powerful inhibitor of human tyrosinase among the 50,000 screened compounds.	[37]

**Table 2:** Physicochemical properties of kojic acid.

Physicochemical Properties		References
Molecular weight	142.1 g/mol	[71,72]
Melting point	151 °C to 154 °C	[72]
Boiling point	401.67 °C at 760 mmHg	[51]
Log P	−2.45	[73]
Ultraviolet absorption spectrum	Range of 280–284 nm	[74]
pH stability	Unstable at pH levels higher than 7	[75]
pKa	7.90–8.03	[76]
Solubility	Soluble in polar solvents like water, ethanol, and ethyl acetate but less soluble in less polar solvents like ether and chloroform	[77]

**Table 3:** Comparison of the most prominent KA derivatives.

KA Derivative	Synthesis	Biological Activity	Benefits over KA	References
KAD	Esterification of KA with palmitic acid at the hydroxyl group of carbon 5 (C-5) and carbon 7 (C-7), both chemically and enzymatically.	Hydrolyzed by dermal esterases, releasing KA that inhibits tyrosinase enzyme.	- More stable in light, heat, oxidation, and metal chelation. - Stable in a pH range of 3–7. - Higher lipophilicity with improved oil solubility.	[75]
KA monooleate and KA monolaurate	Lipase-catalyzed esterification of KA C-5 with oleic and lauric acid.	Tyrosinase inhibitory activity similar to KA.	- KA esters are approximately 15 times more stable. - Safe and nontoxic. - Higher lipophilicity.	[146,147]
Amino acid derivatives	KA tripeptide amide prepared by solid-phase parallel peptide synthesis using Fmoc-Rink amide AM SURE^®^ resin.	Tyrosinase inhibitory activity.	- KA-FWY-NH_2_ proved to be the best compound, with the highest inhibitory activity (IC_50_: 2.2 µM) compared to KA (IC_50_: 94 µM). - Better stability during storage, where they maintained their inhibitory activity for 2 months, whereas KA lost 50% of its activity after 1 week. - Better thermal stability (up to 50 ֯C) and pH stability under acidic and basic conditions.	[148]
Mannich bases	Nucleophilic substitution in a basic medium produced morpholine, piperidine and pyrrolidine derivatives of C-6 of chlorokojic acid.	Tyrosinase inhibitory activity with a 3,4-dichlorobenzyl piperazine-moiety-containing compound showing the highest activity.	- Stronger tyrosinase inhibitory activity with IC_50_ ranging from 86.2 to 362.1 µM, which is much lower than KA (IC_50_: 418.2 µM). - The hydroxymethyl group in some compounds forms a metal complex with copper ions and the enzyme, which is responsible for the higher inhibitory activity.	[81]
Dimeric derivatives	Synthesized by the condensation of kojyl chloride with potassium salts of thiols in DMF to produce thioether derivatives.	Tyrosinase inhibitory and anti-inflammatory activities.	- Dithioether derivatives showed the strongest inhibitory activity. - Sulfide linkage is the critical factor for the inhibitory activity, as it helps the compound to bind well to tyrosinase enzyme, as well as helping to reduce NO production. - The lipophilic alkyl chain can increase the overall lipophilicity of the compound.	[144]

**Table 4:** Advantages and drawbacks of the commonly used nanocarriers in topical delivery.

Nanocarrier	Advantages	Commonly Reported Drawbacks	References
Microemulsions	- Deliver both hydrophilic and lipophilic drugs. - Thermodynamically stable and formed spontaneously. - Improve drug stability and control of release kinetics.	- High concentration of surfactants, which increases skin irritation potential. - Sensitive to temperature, pH, or ionic strength changes. - Complexity of microemulsion formulation.	[151,152]
Nanoemulsions	- Improve rate of absorption into the skin. - Lower surfactant concentration compared to microemulsions, so less potential for skin irritation. - Can be formulated into different dosage forms like creams.	- Thermodynamically unstable. - Oswald ripening. - Low skin adhesion due to their low viscosity.	[153,154,155]
Solid lipid nanoparticles	- Occlusive properties increase skin hydration. - Enhance drug penetration. - Easy to scale up. - Lipids are biocompatible with the skin, so low irritation potential.	- Limited drug loading due to their crystalline nature. - Drug expulsion during storage affects shelf life.	[156,157]
Nanostructured lipid carriers	- Higher loading capacity compared to solid lipid nanoparticles. - Better stability on storage. -Sustained release of drugs.	- Higher amounts of surfactants used cause skin irritation.	[158,159]
Liposomes	- Biocompatible and biodegradable. - Encapsulate both hydrophilic and hydrophobic drugs. - Extended dermal release. - Increase skin moisturization.	- Expensive to prepare. - Structurally unstable, leading to drug leakage.	[160,161]
Ethosomes	- Encapsulate both hydrophilic and hydrophobic drugs. - Higher skin permeability and drug loading capacity compared to liposomes. - Deeper skin delivery.	- Colloidal instability. - Difficult to scale up. - Drug leakage. - Possible skin irritation due to ethanol content.	[162,163]
Niosomes	- Encapsulate both hydrophilic and hydrophobic drugs. - Higher chemical stability compared to liposomes. - Enhanced drug penetration into the skin increases dermal bioavailability. - Biocompatible and non-immunogenic. - Cost-effective.	- Less biocompatible than liposomes due to edge activators. - Concerns regarding physical instability during storage, causing aggregation, sedimentation, or drug leakage.	[164,165]
Spanlastics	- Encapsulate both hydrophilic and hydrophobic drugs. - Highly deformable, improving drug permeation across the skin.	- Manufacturing and stability challenges. - Insufficient reported studies.	[166,167]
Polymeric nanoparticles	- Controlled drug release. - High protection from enzymatic and chemical degradation.	- Long-term toxicity is a concern. - Difficult to scale up.	[168,169,170]
Mesoporous silica nanoparticles	- High surface area. - High loading of both hydrophilic and lipophilic drugs. - Enhance deeper skin permeation. - Tunable to allow stimuli-responsive release. - Biocompatible. - Controlled drug release. - Chemically and thermally stable.	- Surface modification can influence toxicity assessment. - Long-term stability needs to be assessed. - High cost of scaling up.	[164,171]

AChE: acetylcholinesterase; DPPC: dipalmitoyl phosphatidylcholine; DSPC: 1,2-distearoyl-sn-glycero-3-phosphocholine; HPMC: hydroxypropyl methylcellulose; KA: kojic acid; KAD: kojic acid dipalmitate; KAE: kojic acid ester; KMO: kojic acid monooleate; ME: microemulsion; MNPs: magnetic nanoparticles; MSNs: mesoporous silica nanoparticles; N.D.: not disclosed; NE: nanoemulsion; PEG: polyethylene glycol; PLGA: poly(lactic-co-glycolic acid); PS: particle size; SAA: surfactant; W/O/W: water in oil in water; ZP: zeta potential.

## Data Availability

No new data were created or analyzed in this study. Data sharing is not applicable to this article.
